# The Intravenous Injection of Artificial Blood-serum for the Relief of Toxæmia and Extensive Hemorrhage

**Published:** 1897-06

**Authors:** Thomas V. Simpson

**Affiliations:** Yorkton, N. W. T.


### REPORTS OF CASES.

### THE INTRAVENOUS INJECTION OF ARTIFICIAL BLOOD-SERUM FOR THE RELIEF OF TOXÆMIA AND EXTENSIVE HEMORRHAGE.

By Thomas V. Simpson, V.S.,
YORKTON, N. W. T.

It is my desire to call the attention of veterinarians to the use of intravenous or subcutaneous injection of saline solution for the relief of toxæmia, whether it be due to specific microörganisms or to the introduction into the animal economy of poisons from some other source, and to its use in combating extensive hemorrhages from any cause. The most satisfactory results have been obtained in cases of collapse from hemorrhage, the artificial serum not only taking the place of the lost blood, but, according to some writers, having a decided hæmostatic action.

In grave septic infection or intoxication it is clearly indicated. When a large injection has been given for toxæmia there seems to be only very slight improvement for a few minutes; then there develops a strong rapid pulse, rigors, great uneasiness, and the sweat-glands and kidneys pour forth their excretions in large quantities. I have had the opportunity of using saline injections in but one case, and the improvement was so well marked and recovery so quick that I will here give full particulars.

A Clydesdale mare, weighing about thirteen hundred pounds, gave birth to a dead foal. The mare in her struggles had got her head and feet fast under the manger, and had probably been in that position for three or four hours when released by an attendant. She quickly got on her feet, and showed indications of fever. When I arrived I found that she had not ejected the fœtal coverings; pulse was 60 and temperature a little above normal. I gave her a uterine injection of creolin in solution.

This occurred in the morning. At night she was lying down; but pulse and temperature remained high. As she was in good condition I decided to try venesection, and accordingly did so to the amount of eight quarts. This appeared to give relief, the pulse now being quite soft, while before it was hard and full. Next morning I found her a great deal worse; pulse 100 and temperature away up (having broken my thermometer, I guessed the temperature by placing one or two fingers in her mouth). She was lying down and showing well-marked symptoms of septic metritis.

Prognosis doubtful. I then decided, with the consent of owner, to try intravenous injection of salt solution, and accordingly ordered five gallons of water to be boiled. In the meantime thoroughly curetted the uterus, after injecting creolin solution. Then she ejected all the creolin solution, and I gave her an injection of weak iodine solution, which she retained. At 11 A.M. I injected the saline solution into her jugular, taking about forty-five minutes to do so.

In the evening she looked a good deal brighter; temperature not much up, and pulse 80; passes clear urine three or four times an hour. Gave three more gallons of saline fluid intravenously, and clothed heavily to induce perspiration. Also gave another uterine injection of solution of iodine. She would frequently pass a small quantity of dirty-brown putrid matter from her uterus. The next day her pulse and temperature were the same, slight rigors and passage of putrid fluid still taking place. I then gave seven gallons of saline fluid intravenously. Through a little neglect the assistant allowed a little air to enter the injection-tube and thence into the jugular, causing no inconvenience other than increasing the heart's action from 80 to 100 beats per minute. She stood still during the entire operation, but ten minutes after the injection was completed she lay down and seemed in great pain, shivered for a few minutes, and then broke out into a profuse sweat. Breathing was labored and irregular, and the heart's action was 100 and strong. A great deal of brown fluid escaped from the uterus when she lay down. Urine was passed continuously for half an hour. Then she got up and ate a bran-mash. She continued to perspire and urinate freely till next day, and in two more days all the symptoms of toxæmia disappeared.

The solution I used was made by adding one teaspoonful of sodium chloride to a quart of boiled water. The fluid used in the Massachusetts General Hospital is highly recommended, the formula of which is 0.1 gramme (grs. jss) CaCl, 0.75 gramme (grs. x) KCl, to 1000 c.c. (qts. ij) of normal saline solution. The apparatus I adopted consisted of an ordinary household fountain-syringe, to which is attached the required amount of rubber-tubing. The end of the tubing fitted to an ordinary horse-canula. The vein is opened with a lance after being raised, and while still raised the canula is inserted. Care must be taken to see that no air is in the rubber-tube before injecting. Everything must be kept thoroughly scalded with hot water before using. The infusion of saline fluid is indicated in the following conditions: Serious hemorrhage from any cause or hemorrhagic collapse; septicæmia or uræmia in all infectious diseases in which there is a well-marked toxic condition of the blood, tetanus, azoturia, strychnine-poisoning, etc. It has been used with good results in acute pneumonia in man. In all forms of grave poisoning it is clearly indicated. When the infusion is used for the relief of a toxic condition of the blood it should be immediately preceded by venesection.

